# Intra-arterial Bevacizumab in Adult Patients With Steroid-Refractory Cerebral Radiation Necrosis: An Observational Study of Clinical Indications and Outcomes

**DOI:** 10.7759/cureus.80522

**Published:** 2025-03-13

**Authors:** Lisa B Shields, Robert J Kadner, Mahan Ghiassi, Christopher T Shelburne, Michael W Daniels, Shervin R Dashti

**Affiliations:** 1 Neurological Surgery, Norton Neuroscience Institute, Norton Healthcare, Louisville, USA; 2 Neuroradiology, DXP Imaging, 40216, USA; 3 Bioinformatics and Biostatistics, University of Louisville, Louisville, USA; 4 Neurosurgery, Billings Clinic, Billings, USA

**Keywords:** arteriovenous malformation, bevacizumab, brain, brain metastasis, intra-arterial, neurosurgery, radiation necrosis

## Abstract

Background

Cerebral radiation necrosis (RN) is a chronic inflammatory process that may develop following radiotherapy for a primary or metastatic brain tumor or arteriovenous malformation (AVM). A single infusion dose of intra-arterial (IA) bevacizumab (BV) is a viable option. The aim of this study was to assess the safety and efficacy of IA BV infusion in patients with steroid-refractory cerebral RN.

Materials and methods

A total of 33 patients with imaging-confirmed brain RN underwent at least one IA BV infusion over a 9-year duration. BV was administered as a single 2.5 mg/kg (n=24) or 5.0 mg/kg (n=9) infusion dose.

Results

Diagnoses included brain metastasis (17 (51.5%)), AVM (10 (30.3%)), and primary brain tumors (six (18%)). All patients experienced either complete relief or significant symptom improvement after the IA IV infusion. The initial brain MRIs performed following the IA BV infusion revealed a decreased size of the RN in all patients. Two patients had minimal side effects. Of the 16 (48.5%) patients who experienced an RN recurrence, 10 underwent a repeat IA BV infusion. The mean duration between the first IA BV infusion and last follow-up was 23.2 months (range: 0.1-85.2 months). Age (p = 0.00587), tumor pathology (p = 0.03509), and BV dosage (p = 0.02420) were significant predictors of RN recurrence.

Conclusions

IA infusion of BV was well-tolerated by all patients, with substantial clinical and radiological improvement. Further research is needed to explore additional factors that may impact the effectiveness of BV treatment in RN patients.

## Introduction

While radiotherapy offers a promising treatment for primary brain tumors, brain metastatic lesions, and arteriovenous malformations (AVMs) with a prolonged survival benefit, it is also associated with adverse side effects such as radiation necrosis (RN), demyelination, and vascular damage [1, 2]. It has been reported that between 10-50% of patients who undergo stereotactic radiosurgery (SRS) experience cerebral RN, which depends on the total radiation dose, fractionation regimen, and volume of irradiated areas, as well as chemotherapy exposure and anatomic location of the tumor/radiation [1-8]. Radiation causes endothelial cell dysfunction, resulting in capillary permeability, tumor tissue hypoxia, and an increased release of vascular endothelial growth factor (VEGF) [1-5, 7-11]. VEGF is a regulator of angiogenesis which leads to increased vascular permeability, disruption of the blood-brain barrier (BBB), and cerebral edema. Patients may experience neurologic deficits as a direct result of the RN and surrounding edema, or as an indirect result of increased intracranial pressure [1]. 

Although no standard treatment exists for RN, medical management with high-dose corticosteroids, antiplatelet agents, anticoagulants, hyperbaric oxygen therapy, laser interstitial thermal therapy (LITT), high-dose vitamin E, and surgical resection of the necrotic brain tissue to reduce the mass effect are typically used to treat RN [1-5, 7, 9, 10, 12]. While high-dose corticosteroids are the mainstay of care for RN to counteract vascular endothelial damage and relieve cerebral edema, they are often associated with negative side effects (behavioral changes, altered sleep patterns, appetite changes) [1, 2, 9]. The humanized monoclonal anti-VEGF antibody bevacizumab (BV) has been shown to be well-tolerated, safe, and efficacious in treating cerebral RN [1, 3-6, 8, 11, 13, 14]. BV blocks VEGF from reaching its targets on the endothelium, thereby reducing vascular permeability, diminishing BBB damage, mitigating cerebral edema, and improving neurologic symptoms without a dependency on corticosteroids [3, 7, 11, 12, 15]. 

Although RN is a relatively straightforward diagnosis in patients treated for AVM, in tumor patients it is often difficult to differentiate RN from tumor progression; in such cases, a brain biopsy is the diagnostic gold standard [2, 3]. RN may appear as a contrast-enhancing mass with necrotic white matter changes and edema within or adjacent to the site of the original tumor on brain MRI [2, 9]. BV is associated with numerous serious systemic complications including severe hypertension, proteinuria, wound dehiscence, gastrointestinal tract perforation, and vascular events such as intracranial hemorrhage, pulmonary embolus, and sinus thrombosis [4, 5, 7]. 

We report 33 patients who were treated with intra-arterial (IA) BV for radiation-induced necrosis. The study objectives were to assess both the (1) safety and (2) efficacy of IA BV infusion in patients with steroid-refractory cerebral RN. The use of BV to treat primary and metastatic brain tumors and AVMs is discussed. The value of utilizing the IA route in administering BV is also presented.

## Materials and methods

Participants

Under an Institutional Review Board-approved protocol and according to the Declaration of Helsinki, we performed a retrospective review of all patients at our Institution who underwent a targeted IA BV infusion over a 9-year duration (July 13, 2015 - July 19, 2024). All patients underwent salvage RN usual care protocols at our institution. All patients underwent at least one IA BV infusion. Inclusion criteria included adults ≥ 18 years with a Karnofsky Performance Status (KPS) score ≥ 70, who underwent radiotherapy (whole brain radiation therapy (WBRT) or SRS) of the brain, and had a life expectancy ≥ 3 months. The included patients had evidence of increased postcontrast T-1-weighted enhancement with central hypointensity in the irradiated area and confluent increased surrounding vasogenic edema on T2-weighted FLAIR MRI [5]. In addition, RN in an AVM was determined by observing an enhancing lesion on the brain MRI, while a brain biopsy confirmed RN in a brain tumor. To be included in this study, patients had to exhibit at least one symptom of RN (severe headache, recurrent seizures, or neurological deficit) and had to have failed corticosteroid treatment. The exclusion criteria consisted of patients who had a biopsy-proven active malignant brain tumor, active bleeding, a medical condition with a high risk of bleeding, or were consuming anticoagulants other than aspirin [5]. Patients were also excluded if they had an abdominal fistula, abscess, or gastrointestinal tract perforation; undergone a major surgery within 4 weeks of the IA BV infusion; a significant uncontrolled illness; or were pregnant. 

Several metrics were collected, including the patient’s age, gender, age, Body Mass Index (BMI), pathology (AVM versus primary/metastatic brain tumor), angiography without BV prior to angiography with IA BV infusion, radiation and/or IV BV prior to the IA BV infusion, and contraindications to IV BV. We also documented the reasons for IA BV infusion, whether a patient experienced side effects of the IA BV or a recurrence of cerebral RN, the number of IA BV infusions, whether a double dose (5.0 mg/kg) of BV was administered, and the treatment after the initial IA BV infusion. 

Surgical technique of IA BV infusion 

A detailed description of the IA BV infusion procedure has been previously reported [5]. Briefly, patients underwent catheterization of the ipsilateral internal carotid or vertebral artery in our neurointerventional suite. After confirmatory angiography, osmotic blood-brain barrier disruption (BBBD) was conducted with warmed (37°C) 25% mannitol infused at the optimal rate for 30 seconds. IA BV was infused over 10 minutes, while the systolic arterial blood pressure was maintained above 120 mm Hg. Patients were treated with either 2.5 mg/kg or 5.0 mg/kg IA BV. The traditional dose was 2.5 mg/kg, however, certain patients received the 5.0 mg/kg dose if they experienced a recurrence of symptoms post-infusion and were retreated. Patients remained in the transitional care unit on the night of the procedure and were discharged the next day.

Outcomes

**Figure 1:**
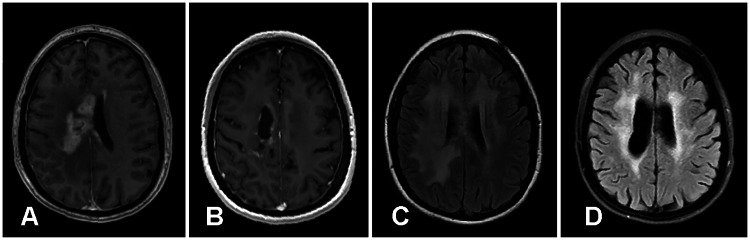
Pretreatment and Posttreatment MRI Scans of the Brain in a Patient with a Hemangiopericytoma (A) Pretreatment MRI scan of the brain.  Axial T1-weighted image obtained following IV gadolinium administration revealing prominent nodular subependymal and parenchymal enhancement, manifest as T1 shortening centered on the lateral and superior walls of the lateral ventricular bodies, right greater than left.  This is located at the site of radiation therapy and represents radiation necrosis. (B) Posttreatment MRI scan of the brain.  Axial T1-weighted image obtained following IV gadolinium administration revealing subtle residual nodular subependymal enhancement, markedly improved compared with pretreatment scan. (C) Pretreatment MRI scan of the brain.  Axial fluid-attenuated inversion recovery (FLAIR) image at the level of treatment, upper lateral ventricles, revealing prominent confluent bilateral white matter edema, manifest as T2 prolongation centered on the upper corona radiata and centrum semiovale, right greater than left.  This is located at the site of radiation therapy and represents vasogenic edema secondary to radiation necrosis. (D) Posttreatment MRI scan of the brain.  Axial FLAIR image revealing subtle residual T2 prolongation, moderately improved compared with pretreatment scan.

**Figure 2:**
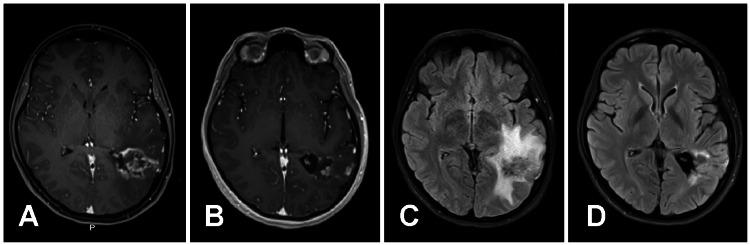
Pretreatment and Posttreatment MRI Scans of the Brain in a Patient with an Arteriovenous Malformation (A) Pretreatment MRI scan of the brain. Axial T1-weighted image obtained following IV gadolinium administration revealing prominent nodular subependymal and parenchymal enhancement, manifest as T1 shortening at the site of stereotactic radiosurgery for treatment of a left temporal lobe arteriovenous malformation (AVM). This image demonstrates the characteristic "cut-green pepper" pattern of enhancement, with an outer edge of linear enhancement within the field of radiotherapy outlining internal hazy striated enhancement. (B) Posttreatment MRI scan of the brain. Axial T1-weighted image obtained following IV gadolinium administration revealing subtle residual nodular subependymal enhancement, markedly improved compared with pretreatment scan. (C) Pretreatment MRI scan of the brain. Axial Fluid-attenuated inversion recovery (FLAIR) image at the level of treatment, trigone of the left lateral ventricle.  This image reveals prominent confluent left temporal white matter edema, manifest as T2 prolongation. This is located at the site of radiation therapy and represents vasogenic edema secondary to radiation necrosis. (D) Posttreatment MRI scan of the brain. Axial FLAIR image revealing subtle residual T2 prolongation, markedly improved compared with pretreatment scan.

Patients were monitored for adverse events for up to 12 months post-infusion. MRI was performed with standard brain-imaging protocol, precontrast and postcontrast sequence, and 1.5-T and 3-T magnets at baseline and at 3- and 12-months post-infusion (Fig. 1A-1D). If patients experienced either worsening clinical symptoms and/or evidence of significant surrounding vasogenic edema and associated mass-effect and brain compression (all consistent with RN) on brain MRIs after the initial IA BV infusion, they were considered for a repeat IA BV infusion or other procedure to treat the brain RN (Fig. 2A-2D). The determination of symptom improvement was made by the treating neurosurgeon who evaluated the patient prior to the IA BV infusion, performed the procedure, and assessed the patient post-procedure. Clinical improvement was dependent on a patient’s particular symptomatology, for example, improvement in either pain or neurological deficit.

Statistical analysis

Several analyses were performed to evaluate the factors influencing RN recurrence and overall survival. These analyses included comparisons between different dosing regimens, BMI groups, pathology types, and RN recurrence status. The analyses were carried out using R software (version 4.3.1; R Foundation for Statistical Computing, Vienna, Austria), employing the following packages: dplyr for data manipulation, tableone for creating descriptive statistics tables, survival for survival analysis and Kaplan-Meier (KM) plots, and kableExtra for table formatting.

Descriptive Statistics and Group Comparisons

Baseline characteristics of patients were summarized and compared across different subgroups using descriptive statistics and Fisher's exact tests for categorical variables, and Wilcoxon rank-sum tests for continuous variables. The subgroups analyzed included BV dosing regimen (standard vs. higher dose), BMI groups (low vs. high), pathology types (tumor vs. AVM), and RN recurrence status.

Kaplan-Meier Survival Analysis

Kaplan-Meier (KM) survival curves were generated to compare the time to RN recurrence between subgroups. Log-rank tests were used to assess the statistical significance of differences in survival distributions.

Logistic Regression Analysis

A logistic regression model was fitted to identify predictors of RN recurrence. The model included age, tumor pathology (tumor vs. AVM), and BV dosing regimen as covariates. The adjusted odds ratios (ORs) with 95% confidence intervals (CIs) and p-values were reported for each predictor.

## Results

Clinical characteristics

A total of 33 patients received at least one IA BV infusion (Table 1). Thirty-one of these patients were diagnosed with steroid-refractory RN. One patient did not have steroid-refractory RN, however, was diagnosed with metastatic renal cell carcinoma and underwent IA BV infusion for brain tumor embolization prior to tumor resection. Another patient also did not have steroid-refractory RN as he had not undergone radiotherapy for his bilateral acoustic neuromas, neurofibromatosis, and vestibular schwannoma but had responded positively to IV BV. The mean age was 51.4 years (range: 19-81 years), and the majority (21 (64%)) of patients were female. The BMI was 29.8 (range: 14.7-53.5). The two most common pathologies treated with IA BV were brain metastasis (17 (51.5%) patients) and AVM (10 (30.3%) patients). The remaining six (18.2%) patients were diagnosed with a primary brain tumor. Non-small cell lung cancer and breast cancer were the most frequent metastatic brain tumors, comprising five patients each. Table 2 reveals significant differences in age (p < 0.001), prior angiogram without BV (p < 0.001), alive status (p = 0.043), and time from first BV to last contact (p = 0.001) between subgroups stratified by pathology. 

**Table 1:** Demographics of Patients Who Underwent Intra-Arterial Bevacizumab Infusion. IA: intra-arterial; BV: bevacizumab; AVM: arteriovenous malformation; IV: intravenous; SRS: stereotactic radiosurgery; WBRT: whole brain radiation therapy ^*^Brain metastasis: non-small cell lung cancer (5), breast (5), renal cell (3), adenocarcinoma of lung (2), esophageal (1), papillary thyroid (1). ^#^Angiography without BV prior to angiography with IA BV: AVM (10) [8: once, 1: twice, 1: three times; embolization of metastatic renal cell carcinoma (1). ^@^Contraindications to IV BV: currently treated with anti-coagulants due to history of pulmonary embolism/atrial fibrillation/deep venous thrombosis/tricuspid regurgitation (one of these patients also had current pharyngeal abscess) (6); history of atrial fibrillation/cerebrovascular accident, not currently treated with anti-coagulants (2); hypotension with intravenous bevacizumab (1); history of chemotherapy-induced thrombocytopenia/anemia (1); partially healed scalp flap (1).

Features	Category	Number of Patients (%), (n=33)
Age		Mean: 51.4 years (Range: 19-81 years)
Gender	Male	12 (36%)
	Female	21 (64%)
Body Mass Index, Kg/m^2^		29.8 (Range: 14.7-53.5)
Pathology	Brain metastasis*	17 (51.5%)
	AVM	10 (30.3%)
	Meningioma	2 (6%)
	Astrocytoma	1 (3%)
	Hemangiopericytoma	1 (3%)
	Mastoid and skull epithelial tumor	1 (3%)
	Acoustic neuroma, neurofibromatosis, vestibular schwannoma	1 (3%)
Angiography without IA BV infusion prior to angiography with IA BV infusion	Yes^#^	11 (33%)
	No	22 (67%)
Radiation prior to IA BV infusion	Yes	31 (94%)
	SRS only	28
	WBRT only	1
	SRS and WBRT	2
	No	2 (6%) (1: brain tumor embolization prior to surgery; 1: previous IV bevacizumab)
IV BV prior to IA BV infusion	Yes	5 (15%)
	No	28 (85%)
Contraindications to IV BV	Yes	11 (33%)
	No^@^	22 (67%)

**Table 2:** Patients Who Underwent Intra-Arterial Bevacizumab Infusion Stratified by Pathology This table presents patient characteristics stratified by pathology type (AVM vs. Tumor) in a cohort of individuals who underwent intra-arterial bevacizumab infusion. Continuous variables, including age, body mass index, and time-related measures, were analyzed using the Wilcoxon rank-sum test. In contrast, categorical variables such as gender, prior angiogram without bevacizumab, standard dosage, radiation necrosis recurrence, and survival status were analyzed using Fisher’s Exact test, with odds ratios (OR) reported where applicable. Significant findings included a lower median age for patients with AVM (27.0 years) compared to those with tumors (62.0 years, p < 0.001). Patients with AVM were significantly more likely to have had a prior angiogram without bevacizumab (100% vs. 4.3%, p < 0.001). The AVM group's survival rate was significantly higher (100% vs. 47.8%, p = 0.005). Time from the first bevacizumab infusion to the last contact was substantially longer in AVM patients (49.0 months vs. 10.0 months, p < 0.001). AVM: Arteriovenous Malformation, BV: Bevacizumab, RN: Radiation Necrosis

Characteristics	Overall (n=33)	AVM (n=10)	Tumor (n=23)	Statistical Test	P-Value
Age, years (median (IQR))	52.0 (44.0, 64.0)	27.0 (21.2, 45.5)	62.0 (48.5, 67.0)	Wilcoxon, W=24	<0.001
Male (n (%))	12 (36.4%)	4 (40.0%)	8 (34.8%)	Fisher’s Exact, OR=3.25 (0.76, 15.74)	1
Body Mass Index, Kg/m2 (median (IQR))	28.2 (24.3, 35.7)	26.4 (24.3, 35.1)	28.4 (23.9, 35.6)	Wilcoxon, W=110	0.845
Prior Angiogram w/o BV (n (%))	11 (33.3%)	10 (100.0%)	1 (4.3%)	Fisher’s Exact, OR=5.13 (0.11, 2.29)	<0.001
Standard Dosage (n (%))	24 (72.7%)	6 (60.0%)	18 (78.3%)	Fisher’s Exact, OR=1.44 (0.34, 6.41)	0.4
RN Recurrence (n (%))	16 (48.5%)	6 (60.0%)	10 (43.5%)	Fisher’s Exact, OR=0.17 (0.02, 0.89)	0.465
Alive (n (%))	21 (63.6%)	10 (100.0%)	11 (47.8%)	Fisher’s Exact, OR=1.54 (0.37, 6.75)	0.005
Time from 1st BV to Last Contact (months) (median (IQR))	20.6 (4.9, 38.8)	49.0 (32.9, 68.9)	10.0 (3.8, 24.2)	Wilcoxon, W=213	<0.001
Time between 1st/2nd BV (weeks) (median (IQR))	8.4 (5.8, 19.6)	16.1 (6.1, 59.1)	7.3 (4.1, 9.6)	Wilcoxon, W=18	0.251
Time between 2nd/3rd BV (weeks) (median (IQR))	9.0 (5.7, 14.9)	20.9 (17.9, 23.8)	5.7 (3.0, 7.4)	Wilcoxon, W=6	0.083

Treatment prior to IA BV infusion

Eleven (33.3%) patients underwent angiography without BV prior to angiography with IA BV infusion, 10 of whom had an AVM (Table 1). A total of 31 (94%) patients were treated with radiotherapy before the IA BV infusion, 30 of whom underwent SRS. Five (15%) patients received IV BV prior to the IA BV infusion. Eleven (33.3%) patients had a contraindication to IV BV; six of these patients were currently treated with anticoagulants due to a history of pulmonary embolism, atrial fibrillation, deep venous thrombosis, and/or tricuspid regurgitation. One patient was treated with LITT prior to the IA BV infusion. 

IA BV infusion

Patients reported a wide array of symptoms that necessitated the IA BV infusion: headaches were most common (15 (45%) patients), followed by weakness/paresthesia/numbness/pain of the extremities (14 (42%) patients), seizures (11 (33%)), and aphasia/worsening memory/cognitive change/confusion/forgetfulness/steroid psychosis (10 (30%)) (Table 3). At the first neurosurgical appointment within 6 weeks of the first IA BV infusion, all patients experienced either a complete relief or significant improvement of symptoms. Additionally, the first brain MRIs performed following the initial IA BV infusion revealed a decrease in size of the RN in all patients. Only two patients had side effects following the IA BV infusion, specifically, one who reported transient increased somnolence and incontinence and another who experienced headaches for several days after the procedure. 

**Table 3:** Treatment and Follow-Up of Patients Who Underwent Intra-Arterial Bevacizumab Infusion IA: intra-arterial; BV: bevacizumab; RN: radiation necrosis *Recurrence of radiation necrosis after the first IA BV infusion: brain MRI showed increasing vasogenic edema (12 patients); clinical symptoms with negative findings on MRI or no MRI performed (3 patients); brain biopsy (1 patient). ^#^Other: craniotomy to remove necrotic tissue (2 patients); two subsequent craniotomies for tumor resection (1 patient); temporal lobectomy/amygdalohippocampectomy for intractable epilepsy (1 patient); angiogram without bevacizumab (1 patient); laser ablation of brain lesion (1 patient); stereotactic radiosurgery (1 patient).

Features	Category	Number of Patients (%), (n=33)
Reason for initial IA BV infusion	Headaches	15 (45%)
	Weakness/paresthesia/numbness/pain of extremities	14 (42%)
	Seizures	11 (33%)
	Aphasia/worsening memory/cognitive change/ confusion/forgetfulness/steroid psychosis	10 (30%)
	Balance problems	2 (6%)
	Dizziness	1 (3%)
	Hearing abnormalities/tinnitus	1 (3%)
	Fatigue	1 (3%)
	Lip quivering	1 (3%)
	Assess for radiation necrosis versus recurrent tumor	1 (3%)
	Brain tumor embolization	1 (3%)
Number of IA BV procedures	1	33 (100%)
	2	10 (30%)
	3	5 (15%)
RN recurrence after initial IA BV infusion	Yes*	16 (48.5%)
	No	17 (51.5%)
Side effects of intra-arterial bevacizumab	No	31 (94%)
	Yes	2 (6%) (1: increased somnolence/incontinence; 1: headaches for several days after procedure)
Double dose (5 mg/kg) of IA BV	No	24 (73%)
	Yes	9 (27%) (2 patients had double doses during two separate IA BV infusion procedures)
Treatment after initial IA BV infusion	None	16 (49%)
	2^nd^ IA BV procedure	10 (30%)
	Other	7 (21%)^#^
Time duration between initial IA BV infusion and last follow-up		Mean: 23.2 months (Range: 0.1-85.3 months)
Time duration between initial IA BV infusion and 2^nd^ IA BV infusion		Mean: 21.0 months (0.1-81.3 months) (0.1 month represented bilateral procedures within 3 days)
Time duration between 2^nd^ IA BV infusion and 3^rd^ IA BV infusion		Mean: 11.3 months (0.2-26.8 months) (0.2 months represented bilateral procedures within 5 days)

All patients underwent at least one IA BV infusion; 10 (30%) patients underwent two procedures, while five (15%) patients had three procedures. Nine patients were treated with a double dose (5.0 mg/kg) of BV; two of these patients had a double dose in two separate IA BV infusions. There was no statistically significant difference between the standard dose and higher dose groups.

Follow-up after the initial IA BV infusion

A total of 16 (48.5%) patients experienced a recurrence of RN after the initial IA BV infusion; 12 had evidence of increasing vasogenic edema on brain MRI, three had clinical symptoms of RN (two had no RN on brain MRI; one did not have a brain MRI), and one had RN detected by a brain biopsy. Of these 16 patients, 10 were treated by a second IA BV infusion, one underwent an angiography without BV, one had a craniotomy to remove the RN, another underwent a laser ablation of the metastatic lesion in the cerebellum, and one underwent two subsequent craniotomies for resection of brain metastasis. Three of the 16 patients did not pursue additional treatment; two of them died within 2 months of the initial IA BV infusion. The mean duration between the first IA BV infusion and last follow-up was 23.2 months (range: 0.1-85.2 months). The mean duration between the first and second IA IV infusions was 21.0 months (range: 0.1-81.3 months); the mean duration between the second and third IA BV infusions was 11.3 months (range: 0.2-26.8 months). At last follow-up, 10 (30%) patients had died (eight with brain metastasis and two with a meningioma). 

Significant differences were observed in age and time from the first BV to last contact between patients with and without RN recurrence (Table 4). Additionally, significant differences were observed in age, prior angiogram without BV, alive status, and time from first BV to last contact between the groups stratified by pathology and BV dosing regimen (Table 5). Table 6 depicts significant differences were observed in age, prior angiogram without BV, alive status, and time from first BV to last contact between the groups stratified by pathology and RN recurrence status. These findings reflect that younger patients and those with AVMs have longer survival times and lower RN recurrence rates compared to older patients and those with tumors. 

**Table 4:** Patients Who Underwent Intra-Arterial Bevacizumab Infusion Stratified by Radiation Necrosis Recurrence This table compares patient characteristics between those with and without radiation necrosis (RN) recurrence after intra-arterial bevacizumab infusion. Wilcoxon rank-sum tests were used for continuous variables such as age, body mass index, and time-related measures. In contrast, Fisher’s Exact test was used for categorical variables, including gender, tumor pathology, prior angiogram without bevacizumab, standard dosage, and survival status. Significant findings included a lower median age in patients with RN recurrence (46.5 years) than those without recurrence (63.0 years, p = 0.004). Patients with RN recurrence had a significantly longer median time from the first bevacizumab infusion to the last contact (31.3 months) than those without recurrence (10.0 months, p = 0.023). No significant differences were observed in gender distribution, body mass index, tumor pathology, prior angiogram, standard dosage, or survival rate. RN: Radiation Necrosis, BV: Bevacizumab

Characteristics	Overall (n=33)	No RN (n=17)	RN (n=16)	Statistical Test	P-Value
Age, years (median (IQR))	52.0 (44.0, 64.0)	63.0 (59.0, 68.0)	46.5 (33.0, 50.0)	Wilcoxon, W=216.5	0.004
Male (n (%))	12 (36.4%)	4 (23.5%)	8 (50.0%)	Fisher’s Exact, OR=3.25 (0.76, 15.74)	0.157
Body Mass Index, Kg/m^2^ (median (IQR))	28.2 (24.3, 35.7)	28.2 (24.5, 35.7)	28.3 (24.0, 35.9)	Wilcoxon, W=133.0	0.914
Tumor Pathology (n (%))	23 (69.7%)	13 (76.5%)	10 (62.5%)	Fisher’s Exact, OR=5.13 (0.11, 2.29)	0.465
Prior Angiogram w/o BV (n (%))	11 (33.3%)	5 (29.4%)	6 (37.5%)	Fisher’s Exact, OR=1.44 (0.34, 6.41)	0.721
Standard Dosage (n (%))	24 (72.7%)	15 (88.2%)	9 (56.2%)	Fisher’s Exact, OR=0.17 (0.02, 0.89)	0.057
Alive (n (%))	21 (63.6%)	10 (58.8%)	11 (68.8%)	Fisher’s Exact, OR=1.54 (0.37, 6.75)	0.721
Time from 1^st^ BV to Last Contact (months) (median (IQR))	20.6 (4.9, 38.8)	10.0 (2.7, 26.7)	31.3 (15.2, 48.6)	Wilcoxon, W=73.0	0.023
Time between 1^st^/2^nd^ BV (weeks) (median (IQR))	8.4 (5.8, 19.6)	6.1 (6.1, 6.1)	9.6 (5.8, 20.7)	Wilcoxon, W=3.0	0.602
Time between 2^nd^/3^rd^ BV (weeks) (median (IQR))	9.0 (5.7, 14.9)	26.8 (26.8, 26.8)	7.4 (4.3, 10.5)	Wilcoxon, W=4.0	0.157

**Table 5:** Patients Who Underwent Intra-Arterial Bevacizumab Infusion Stratified by Pathology and Dosage This table examines patient characteristics based on pathology (AVM vs. tumor) and bevacizumab dosage (Higher Dose vs. Standard Dose). Kruskal-Wallis tests were applied to continuous variables such as age, body mass index, and time-related measures. In contrast, Fisher’s Exact test was used for categorical variables including gender, prior angiogram without bevacizumab, RN recurrence, and survival status. Significant findings included differences in age across the groups (p = 0.003), with the median age being lowest in AVM patients receiving the standard dose (21.5 years) and highest in tumor patients receiving the standard dose (62.5 years). Prior angiograms without bevacizumab were significantly more common in AVM patients (100%) compared to tumor patients (5.6%, p < 0.001). Survival rates were considerably higher in AVM patients receiving the standard dose (100%) and higher doses (100%) compared to tumor patients receiving the standard dose (44.4%, p = 0.023). Time from the first bevacizumab infusion to the last contact also differed significantly across groups (p = 0.002), with AVM patients on the standard dose having the longest median time (53.0 months). In comparison, tumor patients on the standard dose had the shortest (5.6 months). AVM: Arteriovenous Malformation, BV: Bevacizumab, RN: Radiation Necrosis

Characteristics	Overall (n=33)	AVM: Higher Dose (n=4)	Tumor: Higher Dose (n=5)	AVM: Standard Dose (n=6)	Tumor: Standard Dose (n=18)	Statistical Test	P-Value
Age, years (median (IQR))	52.0 (44.0, 64.0)	44.0 (37.5, 50.0)	50.0 (47.0, 64.0)	21.5 (19.5, 25.8)	62.5 (50.5, 67.5)	Kruskal-Wallis, χ^2^ =13.9	0.003
Male (n (%))	12 (36.4%)	2 (50.0%)	3 (60.0%)	2 (33.3%)	5 (27.8%)	Fisher’s Exact	0.568
Body Mass Index, Kg/m^2^ (median (IQR))	28.2 (24.3, 35.7)	24.5 (22.6, 26.7)	26.9 (25.8, 31.8)	32.0 (25.1, 38.4)	29.3 (23.4, 35.7)	Kruskal-Wallis, χ^2^=2.3	0.513
Prior Angiogram w/o BV (n (%))	11 (33.3%)	4 (100.0%)	0 (0.0%)	6 (100.0%)	1 (5.6%)	Fisher’s Exact	<0.001
RN Recurrence (n (%))	16 (48.5%)	3 (75.0%)	4 (80.0%)	3 (50.0%)	6 (33.3%)	Fisher’s Exact	0.205
Alive (n (%))	21 (63.6%)	4 (100.0%)	3 (60.0%)	6 (100.0%)	8 (44.4%)	Fisher’s Exact	0.023
Time from 1^st^ BV to Last Contact (months) (median (IQR))	20.6 (4.9, 38.8)	47.8 (42.5, 54.5)	20.6 (16.1, 27.7)	53.0 (32.4, 80.7)	5.6 (3.8, 16.2)	Kruskal-Wallis, χ^2^=15.2	0.002
Time between 1^st^/2^nd^ BV (weeks) (median (IQR))	8.4 (5.8, 19.6)	11.1 (6.0, 26.9)	8.4 (5.5, 12.4)	81.3 (81.3, 81.3)	4.1 (4.1, 4.1)	Kruskal-Wallis, χ^2^=3.7	0.298
Time between 2^nd^/3^rd^ BV (weeks) (median (IQR))	9.0 (5.7, 14.9)	20.9 (17.9, 23.8)	5.7 (3.0, 7.4)	NA (NA, NA)	NA (NA, NA)	Kruskal-Wallis, χ^2^=3.0	0.083

**Table 6:** Patients Who Underwent Intra-Arterial Bevacizumab Infusion Stratified by Pathology and Radiation Necrosis Recurrence This table explores patient differences based on pathology (AVM vs. Tumor) and radiation necrosis recurrence (RN vs. No RN). Kruskal-Wallis tests were used for continuous variables, including age, body mass index, and time-related measures. In contrast, Fisher’s Exact test was applied to categorical variables such as gender, prior angiogram without bevacizumab, standard dosage, and survival status. Significant findings included a difference in age among groups (p < 0.001), with AVM patients with RN recurrence having the youngest median age (24.5 years) and tumor patients without RN recurrence having the oldest (65.0 years). Prior angiograms without bevacizumab were significantly more frequent in AVM patients (100%) compared to tumor patients (0%, p < 0.001). Survival rates differed substantially across groups (p = 0.043), with AVM patients in both RN and No RN groups having 100% survival, whereas survival was lower in tumor patients. Time from the first bevacizumab infusion to last contact was also significantly different across groups (p = 0.001), with AVM patients with RN recurrence having the longest time (61.4 months) compared to tumor patients without RN recurrence (4.9 months). RN: Radiation Necrosis, AVM: Arteriovenous Malformation, BV: Bevacizumab

Characteristics	Overall (n=33)	No RN:AVM (n=4)	RN:AVM (n=6)	No RN:Tumor (n=13)	RN:Tumor (n=10)	Statistical Test	P-Value
Age, years (median (IQR))	52.0 (44.0, 64.0)	40.0 (25.0, 54.5)	24.5 (21.2, 37.5)	65.0 (62.0, 70.0)	48.5 (46.2, 51.5)	Kruskal-Wallis, χ^2^ =18.7	<0.001
Male (n (%))	12 (36.4%)	1 (25.0%)	3 (50.0%)	3 (23.1%)	5 (50.0%)	Fisher’s Exact	0.506
Body Mass Index, Kg/m^2^ (median (IQR))	28.2 (24.3, 35.7)	32.4 (25.5, 40.9)	25.7 (23.5, 28.9)	28.2 (20.7, 31.9)	31.6 (25.0, 36.5)	Kruskal-Wallis, χ^2^ =2.1	0.542
Prior Angiogram w/o BV (n (%))	11 (33.3%)	4 (100.0%)	6 (100.0%)	1 (7.7%)	0 (0.0%)	Fisher’s Exact	<0.001
Standard Dosage (n (%))	24 (72.7%)	3 (75.0%)	3 (50.0%)	12 (92.3%)	6 (60.0%)	Fisher’s Exact	0.143
Alive (n (%))	21 (63.6%)	4 (100.0%)	6 (100.0%)	6 (46.2%)	5 (50.0%)	Fisher’s Exact	0.043
Time from 1^st^ BV to Last Contact (months) (median (IQR))	20.6 (4.9, 38.8)	37.0 (26.4, 49.5)	61.4 (48.6, 84.9)	4.9 (1.9, 15.1)	18.3 (7.0, 32.8)	Kruskal-Wallis, χ^2^ =17.7	0.001
Time between 1^st^/2^nd^ BV (weeks) (median (IQR))	8.4 (5.8, 19.6)	6.1 (6.1, 6.1)	37.6 (13.5, 64.7)	NA (NA, NA)	7.3 (4.1, 9.6)	Kruskal-Wallis, χ^2^ =2.2	0.326
Time between 2^nd^/3^rd^ BV (weeks) (median (IQR))	9.0 (5.7, 14.9)	26.8 (26.8, 26.8)	14.9 (14.9, 14.9)	NA (NA, NA)	5.7 (3.0, 7.4)	Kruskal-Wallis, χ^2^ =3.2	0.202

Age (OR = 0.84, 95% CI: 0.74 to 0.95, p = 0.00587), tumor pathology (OR = 138.65, 95% CI: 1.36 to 14108.16, p = 0.03509), and BV dosage (OR = 0.011, 95% CI: 0.0001 to 0.58, p = 0.02420) were significant predictors of RN recurrence. These findings reflect that older age is associated with a lower likelihood of RN recurrence, patients with tumors have a substantially increased likelihood of RN recurrence, and the standard dose of BV is more effective in reducing RN recurrence rates. Kaplan-Meier plots for overall survival did not demonstrate increased survival probability by either BV dose (standard vs higher dose) (p = 0.93) or by BMI (low vs high BMI with a median cutoff of 28.3) (p = 0.616).

## Discussion

Several studies have reported the use of IV BV in treating RN in primary and metastatic brain tumors [1, 3, 6, 7, 9-13, 15-22]. In 2007, Gonzalez and colleagues were the first to report utilizing IV BV in eight patients with malignant brain tumors who developed RN [16]. All patients demonstrated a reduction in both MRI FLAIR abnormalities and T1-weighted post-Gd-contrast abnormalities. There was also a decline in daily dexamethasone consumption. In Khan and colleagues’ systematic review and meta-analysis of 89 patients treated with IV BV for brain RN, 83 (93%) patients recorded a radiographic response to BV, while six (6.7%) had progressive disease [12]. Seventy-three patients demonstrated mean volume reductions on gadolinium-enhanced T2 and T2-weighted fluid-attenuated inversion recovery (FLAIR) MRIs. A total of 85 patients presented with neurological symptoms due to RN (headaches, limb weaknesses, cognitive function, gait problems). After IV BV, nine (10%) patients had stable symptoms, 39 (48%) had improved, and 34 (40%) patients had complete resolution of their symptoms. Dexamethasone was discontinued or reduced in 30 (97%) of 31 patients who had a recorded dosage before and after BV. Only one study in Khan and colleagues’ review reported a recurrence rate; 10 of 13 responders had an RN recurrence [12, 21]. Five studies (63 patients) reported adverse events after IV BV, with hypertension and mild allergy as the most common. The most common dose of IV BV was 5-10 mg/kg, and the mean number of treatment cycles completed ranged from 2-6 cycles. Follow-up ranged from 3.3 to 22.7 months. Khan and colleagues concluded that IV BV provides a radiographic response and clinical improvement without any serious adverse events [12]. 

In Delishaj and colleagues’ systematic review of 125 patients in 21 studies treated with IV BV for brain RN, low-dose IV BV was effective in improving both the clinical (114 [91.2%] patients) and radiographic (122 [97.6%] patients) response [2]. Similar to Khan et al., the most common adverse event of IV BV was hypertension, and the majority (97%) of patients reduced their steroid use after IV BV. The most frequent IV BV dose was 7.5 mg/kg for 2 weeks with a median of four cycles. The median follow-up was 8 months. In Liao and colleagues’ systematic review of 236 patients in 12 studies who received IV BV for brain RN, clinical outcomes and cognitive function were improved in most patients [13]. Neurocognitive improvement was significantly better after 2 months of treatment in patients who received IV BV than in those given corticosteroids. A total of 46 (34%) responding patients (34%) had a recurrence. 

While most studies in the extant literature treated patients with more than one cycle of IV BV for primary and metastatic brain tumors with RN, Voss and colleagues reported one administration of IV BV in 11 patients (11: gliomas, one: breast cancer brain metastasis) [8]. Eight patients received 7.5 mg/kg; three patients received 10 mg/kg. Nine of 10 patients who underwent a brain MRI had marked reduction of edema at first follow-up. Dexamethasone was discontinued in six patients, while the other had a significant dose reduction. One patient sustained a pulmonary artery embolism 2 months after being treated with IV BV. The median time to treatment failure of any cause was 3 months. 

Approximately 20% of patients with an AVM cannot undergo safe and effective surgical intervention due to the size, location, and characteristics of the AVM such as a large nidus, deep venous drainage, and eloquent location [14]. Therefore, SRS is an attractive therapeutic option for these patients [23]. While high-radiation doses are utilized to increase AVM obliteration rates, complications such as hemorrhage and RN may ensue [24]. RN-induced side effects depend on the anatomic location of the AVM and include headaches, paralysis, and ataxia [24]. A high expression of VEGF has been reported in brain AVMs [25]. Few studies have described the use of IV BV in AVMs, either to treat the AVM itself [14, 25] or the cerebral necrosis induced by radiotherapy of the AVM [24, 26-28]. Patients with an AVM treated with IV BV attained significant improvement of symptoms and radiological findings without adverse events [14, 24, 26-28]. The RN associated with post-SRS treated AVMs improves with concomitant symptomatic improvement. 

IA BV delivery has been used in the past in the management of glioblastoma multiforme (GBM) [29]. This was performed via superselective delivery of the drug to the arteries supplying the tumor, resulting in a narrow volume of distribution. In our opinion, this approach misses the mark. In a patient with GBM, it is known that there are malignant tumor cells infiltrating well beyond the confines of the visible enhancing mass. It stands to reason that performing a less selective delivery of BV to the entire hemisphere would be more efficacious. This is the rationale for our approach involving administration of BV in a less selective manner into the appropriate large artery (internal carotid or vertebral arteries or both). Our aim was the delivery of BV to the RN and all of the surrounding brain containing vasogenic edema.

We have previously reported the IA delivery of BV for RN in patients with metastatic brain tumors and AVMs [4, 5]. To minimize the systemic toxicity of IV BV, a single IA infusion of 2.5 mg/kg BV was administered. In our case series with two pediatric patients with cerebral AVMs who developed RN after SRS, the patients had significant and durable clinical radiographic response with a mean follow-up duration of 8.5 months. Both patients attained resolution of their intractable headaches and were weaned off steroids. There was also a greater than 70% reduction in cerebral edema. In our phase II trial (NCT02819479) of 10 patients (eight: AVM, one: meningioma, one: metastatic non-small cell lung cancer (NSCLC)) with cerebral RN who were treated with IA BV, RN volume decreased by 74%, edema volume decreased by 50%, and headaches decreased by 84% among eight patients without RN recurrence at 12 months. No patients had been treated with IV BV prior to the IA BV infusion. Two patients experienced RN recurrence and underwent further medical or surgical treatment at 10 and 11 months, respectively, after BV infusion. There were no adverse events attributed to BV alone. 

Strengths and limitations of the current study

Our present study with 33 patients treated with IA BV for cerebral RN is the largest, to our knowledge, in the literature. All patients experienced significant clinical and radiological improvement following the initial IA BV infusion. No patients reported serious adverse events, which is common with IV BV. However, this positive attribute of IA BV had a trade-off: a high number of RN recurrences in approximately half of the patients. Almost one-third of patients underwent a second IA BV infusion. IA BV is a valuable alternative for patients who have a contraindication to IV BV, as one-third of patients in the current study were unable to be treated with IV BV. Limitations of the present study include its retrospective nature, the small number of patients, and the heterogeneity of the patients’ underlying brain lesion. Additional limitations are the case series level data, which was subject to bias.

## Conclusions

Cerebral RN is an inflammatory process that may ensue after radiotherapy for a primary or metastatic brain tumor or AVM. A single infusion dose of IA BV is a safe and efficacious option to treat cerebral RN. Our study highlighted 33 patients with imaging-confirmed brain RN who underwent at least one IA BV infusion over a 9-year duration. IA infusion of BV was well-tolerated by all patients in our study, with significant clinical and radiological improvement for a mean duration of 23 months. All patients experienced either complete relief or significant symptom improvement after the IA IV infusion. Age, tumor pathology, and BV dosage were significant predictors of RN recurrence. Prospective, randomized studies are warranted to evaluate the efficacy of IA BV in treating cerebral RN in patients with primary and metastatic brain tumors and AVMs. 
